# Fibre Bragg Gratings in Embedded Microstructured Optical Fibres Allow Distinguishing between Symmetric and Anti-Symmetric Lamb Waves in Carbon Fibre Reinforced Composites

**DOI:** 10.3390/s17091948

**Published:** 2017-08-24

**Authors:** Ben De Pauw, Sidney Goossens, Thomas Geernaert, Dimitrios Habas, Hugo Thienpont, Francis Berghmans

**Affiliations:** 1 Department of Applied Physics and Photonics (TONA), Vrije Universiteit Brussel, Brussels Photonics (B-PHOT), Pleinlaan 2, 1050 Brussels, Belgium; sidney.goossens@vub.be (S.G.); tgeernae@vub.be (T.G.); hthienpo@vub.be (H.T.); francis.berghmans@vub.be (F.B.); 2 Flanders Make, Oude Diestersebaan 133, 3920 Lommel, Belgium; 3 Hellenic Aerospace Industry, Engineering Research Design and Development Directorate, 32009 Shimatari, Greece; habas.dimitrios@haicorp.com

**Keywords:** fibre Bragg gratings, Lamb waves, CFRP, impact damages

## Abstract

Conventional contact sensors used for Lamb wave-based ultrasonic inspection, such as piezo-electric transducers, measure omnidirectional strain and do not allow distinguishing between fundamental symmetric and anti-symmetric modes. In this paper, we show that the use of a single fibre Bragg grating created in a dedicated microstructured optical fibre allows one to directly make the distinction between these fundamental Lamb wave modes. This feature stems from the different sensitivities of the microstructured fibre to axial and transverse strain. We fabricated carbon fibre-reinforced polymer panels equipped with embedded microstructured optical fibre sensors and experimentally demonstrated the strain waves associated with the propagating Lamb waves in both the axial and transverse directions of the optical fibre.

## 1. Introduction

Lamb wave-based ultrasonic inspection for damage identification in engineering structures is considered to be an attractive alternative for conventional and time-consuming point-by-point ultrasonic inspection techniques, mainly because Lamb waves can travel long distances across the structure and therefore allow for inspecting fairly large areas. Such Lamb waves can be excited and detected in various manners depending on the size and material of the structure under test [1,2,3]. In the aerospace industry, for example, the most common technique to both excite and detect Lamb waves in plate-like composite components uses piezo-electric transducers (PZTs). Such transducers feature a small size and low power consumption. The main disadvantages of PZTs in Lamb wave-based inspection are twofold. First, each PZT requires being individually connected, which calls for dealing with a multitude of wires when using arrays of transducers. Second, PZTs exhibit unselective sensitivity in the sense that Lamb waves propagating along different directions in the plane of a panel are all picked up with the same sensitivity. Critical directions, corresponding to, for example, a defect in the panel, are recorded simultaneously and with the same sensitivity as every other direction. It is thus challenging to identify the result of a defect in a panel on Lamb waves propagating in this structure and, therefore, it is difficult to locate and assess the size of the defect. Optical fibre Bragg gratings (FBGs) have already been proposed to detect Lamb waves in order to cope with the aforementioned shortcomings [3,4,5,6]. Multiple FBGs can easily be multiplexed on a single optical fibre, which drastically decreases the amount of wiring when using an array of such sensors. Furthermore, and owing to the geometry of the FBG, the response to Lamb waves depends on the angle between the fibre and the Lamb wave’s propagation direction. Finally, the small size of the optical fibre allows these sensors to be embedded between the laminar layers of a composite structure in a minimally intrusive manner, avoiding the need to attach bulky and disturbing sensors to the surface of a structure [7].

Lamb waves can be categorised into two groups based on the associated propagating perturbation of the composite with respect to the mid-plane of a panel, i.e., in symmetric and anti-symmetric modes. These modes are denoted as S_i_ and A_i_, respectively, where i refers to the order of the mode. The number of symmetric and anti-symmetric modes that can propagate along a plate-like structure under test depends on the excitation frequency [3,8]. Owing to their low dispersion and attenuation, the fundamental symmetric S_0_ and anti-symmetric A_0_ modes are frequently considered suitable for inspecting the structure for the presence of defects. Since they have different mode shapes, S_0_ and A_0_ modes are sensitive to different defects in the structure: the S_0_ wave is extensional in nature and thus more sensitive to defects inside the structure under test, whilst the A_0_ is flexural and thus more sensitive to surface cracks, as an example. Both modes also have a different frequency-dependent propagation velocities. Consequently, both types of waves are of interest for identifying and locating damage in composites. However, properly distinguishing between A_0_ and S_0_ waves in the sensor responses remains very difficult in ultrasonic inspection using both conventional FBGs and PZTs [3,9,10,11]. In this paper, we demonstrate that a sensor consisting of a single FBG created in a specialty microstructured optical fibre (so-called MOFBG) can be used to discern between the A_0_ and S_0_ waves. To do so, we used MOFBG sensors embedded within the composite panel to measure the Lamb waves, while until now mostly surface mounted FBGs have been used for this purpose. 

Our paper is structured as follows. Section 2 describes the unique measurement strategy that MOFBG sensors can offer to record Lamb waves. We discuss how the mounting procedure and experimental setup help to distinguish between the A_0_ and S_0_ waves. The subsequent Section 3 deals with the experimental results obtained and discusses how these can be applied when detecting impact damage using Lamb waves. Section 4 closes our paper with a summary and conclusions.

## 2. MOFBG Sensors and Measurement Strategy

**Figure 1:**
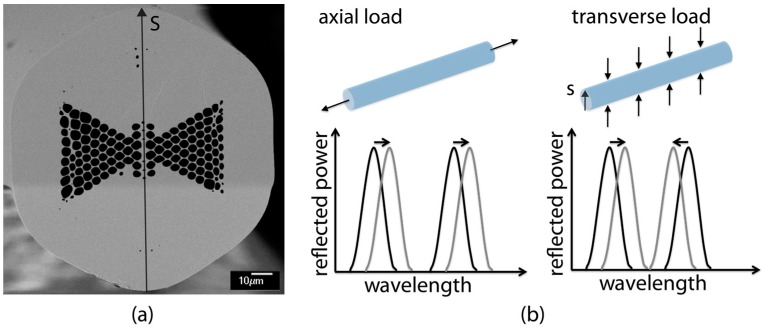
(**a**) SEM image of the cross-section of the fabricated butterfly microstructured fibre (MOF); (**b**) Response of a fibre Bragg grating (FBG) created in the butterfly MOF: axial strain corresponds to both peaks moving in the same way, whilst transverse strain (applied parallel to axis ‘S’) corresponds to the peaks moving in opposite directions [18].

MOFBGs can exhibit peculiar characteristics that cannot be achieved using conventional optical fibre technology. More specifically, the design flexibility of such microstructured fibres (MOFs) allows for the development of sensors that exhibit selective sensitivities to e.g., axial strain, transverse strain, or even shear strain, whilst being negligibly cross-sensitive to temperature changes. Examples of MOF-based sensors have been reviewed in several research papers [12,13,14,15,16,17,18,19,20]. In this paper, we use a specific microstructured fibre design which we refer to as a ‘butterfly’ MOF [17,18,19,20]. Figure 1a shows an SEM image of the cross-section of the butterfly MOF [18]. The peculiar microstructure induces a high level of birefringence in the optical fibre. When this MOF is equipped with an FBG, the latter reflects two Bragg wavelengths corresponding to the two orthogonal polarised propagation modes. The Bragg wavelengths (or peaks) will shift with the same amount when an axial load is applied to the optical fibre, but will move in opposite ways when a transverse load (parallel to axis ‘S’) is applied. The absolute and relative position of the Bragg peaks therefore encodes axial strain as well as transverse strain, respectively, into the reflection spectrum of the MOFBG. This principle is summarised in Figure 1b.

**Figure 2:**
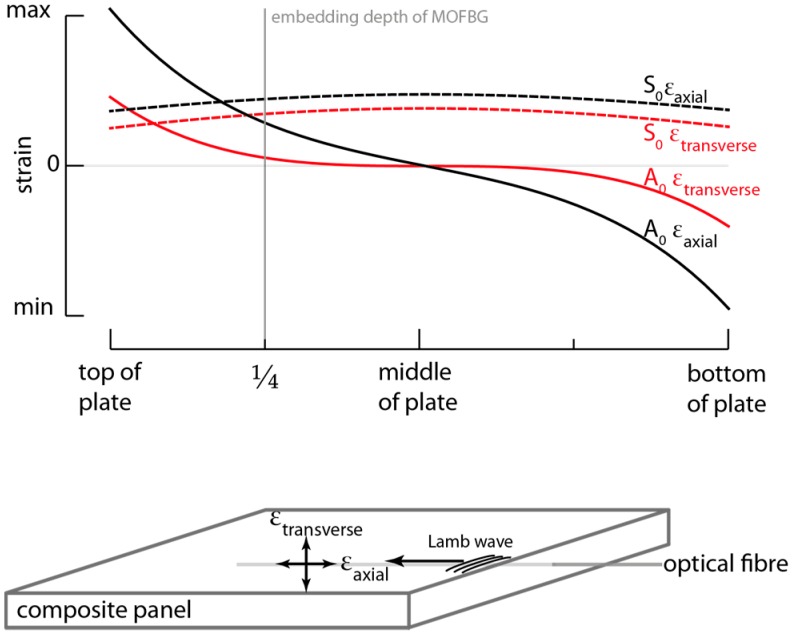
Illustration of strain mode shapes of both S_0_ and A_0_ waves in the lateral direction of a plate-like structure. The strain induced by an A_0_ wave close to the mid-thickness tends to be zero, in contrast to the induced strain of the S_0_ wave, which increases at the same depth. An MOFBG embedded at a depth of, for example, ¼ of the panel thickness, that is sensitive to both axial and transverse strain, can exploit this difference to distinguish between the S_0_ and A_0_ modes [18,21].

The selective sensitivity of a single MOFBG to axial and transverse strain can be exploited to differentiate between the S_0_ and A_0_ modes. This stems from the nature of the induced strain distribution (or strain mode shape) in the axial and transverse directions of a plate-like structure, which is different for these modes. Owing to the anti-symmetric shape of the A_0_, the associated strain amplitude in the transverse direction is close to zero near the mid-plane of the panel [21]. The symmetry of the S_0_ mode, on the other hand, results in an increase of the strain amplitude at that location. Consequently, an MOFBG embedded for example at a depth of ¼ (or equivalently ¾) of the thickness of a plate-like structure will be able to pick up both the S_0_ and A_0_ mode in the axial direction, but mostly the S_0_ mode in the transverse direction, as depicted in Figure 2, which illustrates typical strain mode shapes in the lateral direction of a plate-like structure. At ¼ or ¾ of the thickness, the A_0_ wave as well as the S_0_ wave will still exhibit some transverse strain components, but given the contrast with the axial strain components, the MOFBG allows distinguishing between the two parties. Note that the actual measured strain amplitude of both the S_0_ and A_0_ modes also depends on the transfer of the strain induced in the material by the Lamb waves to the optical fibre and on the excitation amplitude and attenuation of each mode.

**Figure 3:**
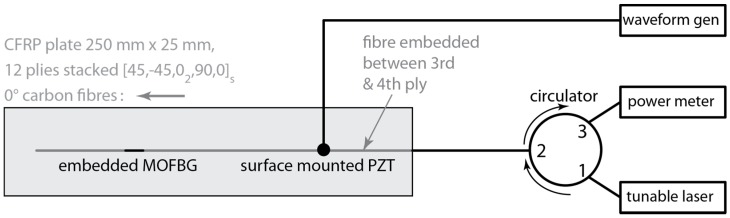
A schematic of the experimental setup.

To demonstrate the capability of an MOFBG to distinguish between S_0_ and A_0_ modes using the approach outlined above, we fabricated a 250 mm by 25 mm carbon fibre-reinforced polymer (CFRP) panel made from Hexcel M21/T800S prepreg, with a lay-up of (45/−45/0_2_/90/0)_s_. The thickness of each ply is close to 0.184 mm, resulting in an overall thickness of 2.21 mm. We embedded an MOFBG between the third and fourth laminar layers (i.e., at ¼ of the thickness of the panel) and aligned the optical fibre with the 0° orientation of the plies (i.e., parallel with the carbon fibres in the plane of the laminate plies) to minimize the effect of the embedding and, in particular, to avoid the creation of resin pockets [7]. We positioned the MOFBG at the half-length of the panel and angularly oriented the MOFBG such that the peak separation was essentially sensitive to the out-of-plane transverse load. To do so, we rotated the fibre about its axis (i.e., changed the roll angle) so that the butterfly axis (referred to as ‘S’ in Figure 1a) was aligned with the desired axis of the CFRP panel. To obtain the desired orientation of the microstructured optical fibre, we relied on our technique described in Reference [18], which yields angular accuracies of the roll angle of the fibre with respect to the instrumented structure close to ±8°. We have previously determined that such a tolerance on the roll angle guarantees a sensitivity to loads along the desired CFRP panel axis of well beyond 85% of the sensitivity achieved when the fibre’s microstructure is perfectly aligned along the desired axis [19]. This is not only sufficient for the demonstration purpose reported in this paper, but also allows one to envisage its implementation in practical applications. To excite the Lamb waves in the fabricated panel, we surface-mounted a disk-shaped PZT of type PIC255 (PI Ceramic GmbH, Lederhose, Germany) with a diameter of 10 mm on one extremity. One important remark here is that the distance between the PZT and the MOFBG is only 10 cm, and is thus typically insufficient for the A_0_ and S_0_ modes to fully separate owing to their different propagation velocities (in the used material). More specifically, we observed from the experimentally and numerically determined dispersion curves of our setup that the delay between the A_0_ and S_0_ waves was only about 18 µs. For the frequency-thickness used in this paper, the measured phase velocities of the S_0_ and A_0_ waves were close to 4.5 m/ms and 2.8 m/ms, respectively. A schematic of the experimental setup is depicted in Figure 3. We controlled the PZT using a waveform generator that generates five-cycle tonebursts with a Hanning filter applied at a preset frequency. The response of the FBG to the impinging Lamb waves was measured using the so-called edge filtering method [22], in which the output wavelength of a tunable laser is tuned to the wavelength where the slope of the Bragg peak is steepest. A shift of the Bragg peak resulting from a passing strain wave is then recorded as a change of optical intensity by a fast photodetector (Thorlabs PDA20CS). Note that the birefringence in the MOFBG produces two Bragg peaks (see Figure 1), meaning that a total of four slopes (two ascending and two descending) can be used.

## 3. Experimental Results

### 3.1. MOFBGs Allow Distinguishing between A_0_ and S_0_ Waves

**Figure 4:**
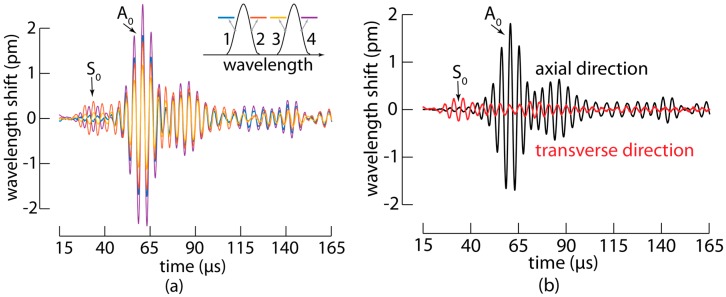
(**a**) The measured wavelength shifts for the four slopes of the MOFBG embedded in the carbon fibre reinforced polymer panel versus time; (**b**) Based on the properties of the MOFBG, the measured signals of the four slopes have been converted into axial and transverse wavelength shifts that are proportional to the respective strains applied to the MOFBG.

Using the setup described above, we excited the fundamental S_0_ and A_0_ modes in the fabricated panel by actuating the PZT with a 20 Vpp five-cycle toneburst at 250 kHz with the amplitude modulated using a Hanning filter, and we measured the response with the embedded MOFBG. To improve the signal-to-noise ratio, each measurement was repeated 1000 times and averaged. The measured wavelength shifts as a function of time for the four slopes of the MOFBG are shown in Figure 4a. The average wavelength shift of the four measurements is proportional to the axial strain applied to the MOFBG, while the shift in spectral distance between the first and third slopes or between the second and fourth slopes is proportional to the transverse strain applied to the MOFBG. The resulting wavelength shifts corresponding to the axial (in black) and transverse (in red) directions are shown in Figure 4b. Although the sensitivity in the transverse direction is not as high as that in the axial direction [18,19,20], we observed a clear difference in the obtained signals. In particular, the signal measured in the axial direction proved to be more responsive to an impinging A_0_ wave (because the PZT excitation of A_0_ is more pronounced), while the signal measured in the transverse direction was more responsive to the S_0_ wave.

The identification of both types of waves is confirmed by the time-of-arrival of both the wave packets that arrive first as well as the wave packets returning after a reflection at the extremity of the panel (see also Section 3.3). The results therefore evidence that a single embedded MOFBG, sensitive to both axial and transverse strains, allows distinguishing between the A_0_ and S_0_ Lamb waves propagating through the CFRP panel.

### 3.2. Selection of Preferential Directions Allow Filtering out Undesired Edge Reflections

**Figure 5:**
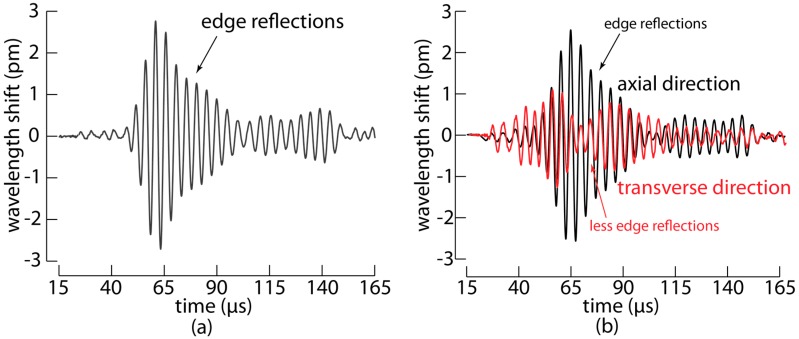
(**a**) A measurement of a single slope of a MOFBG in a similar CFRP panel, where the MOFBG is oriented to be sensitive to axial load and transverse in-plane load; (**b**) The measured responses of the MOFBG converted into axial and transverse loads.

The directional sensitivity of the MOFBG can bring an additional advantage to Lamb wave-based ultrasonic inspection. In a geometry similar to the CFRP panel explained in the previous section, the response of a PZT sensor surface mounted at the position of the embedded MOFBG would be significantly influenced by the multitude of reflections. Such a situation would make it very challenging to distinguish the A_0_ and S_0_ waves from PZT recordings, since a PZT is sensitive to all incident directions and the reflected Lamb waves arrive at the sensor quasi-simultaneous. The key feature of MOFBGs is that they are essentially sensitive to two perpendicular directions (one axial and one transverse to the optical fibre), which allowed us to choose, by carefully angularly orienting the MOFBG, to filter out part of the edge reflections. To demonstrate this, we fabricated a second CFRP panel with the same geometry as before and with the MOFBG embedded at the same location. In this panel, however, we angularly oriented the MOFBG (i.e., adjusted the roll angle of the fibre to align the axis ‘S’ of the butterfly structure) so that to the peak separation is essentially sensitive to the transverse in-plane load. We repeated the measurement procedure and we separated again the axial load and transverse loads, as performed for the previous panel. Figure 5 shows the results. The reflections from the long edges are more clearly observed than in the previous composite panel, as shown in Figure 5a, using a measurement on a single slope. Similar to the previous panel, we noticed a more significant response to the S_0_ wave than to the A_0_ mode in the transverse direction and vice versa in the axial direction. Therefore, carefully aligning the butterfly structure by adjusting the roll angle of the MOFBG during mounting allows for the selection or elimination of certain directions of strain and, hence, potentially accounts for undesired effects resulting from Lamb wave reflections from the edges of a panel under test.

### 3.3. Consequences for Impact Damage Detection in CFRP

The demonstrated ability to resolve the two components of Lamb waves is also beneficial when Lamb waves are used to detect damage in a structure. In most experiments involving Lamb wave-based damage detection, the signals of an undamaged structure are compared to the signals of the damaged structure [23,24,25]. Damage in the structure will cause propagating Lamb waves to reflect and refract, yielding an alteration in the recorded Lamb waves. In general, one can distinguish between two situations. 

**Figure 6:**
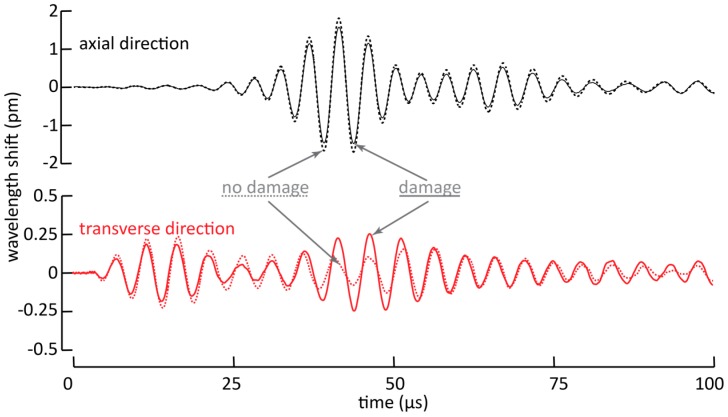
Wavelength shifts corresponding to axial and transverse strains for a pristine and damaged situation for the sample described in Section 3.1.

First, the damage can be on the direct path between the actuator and the sensor. In this case, the fraction of the Lamb waves that are influenced (because of reflection, refraction, attenuation, etc.) will alter the energy contained in the wave packets that are picked up by the sensor [25]. As an example of such a situation, we experimentally mimicked an impact damage by applying a dot of synthetic rubber adhesive with a diameter of about 5 mm to the CFRP panel described in Section 3.1, and we recorded Lamb waves with the embedded MOFBG. We show the result in Figure 6, in which we have plotted the wavelength shifts corresponding to strains in the axial and transverse directions. The dashed lines in the figure indicate the pristine situation, while the solid lines correspond to the damaged situation. Since the simulated damage is located on the direct path between the PZT actuator and the MOFBG sensor, a fraction of the Lamb waves will be attenuated, yielding less energetic strain waves to be transmitted and thus smaller recorded wavelength shifts. The latter can be clearly seen in both the axial and transverse directions when the first wave packets arrive at the MOFBG. In addition, due to the additional attenuation which is possibly accompanied with partial mode conversion from A_0_ to S_0_, the second wave packet shows a significantly larger amplitude compared to the undamaged state in the transverse direction. In the described situation, we observed an increase of the amplitude of the second wave packet of more than a factor of 2. Recall, however, that for the used setup the excitation of the A_0_ mode is more pronounced compared to the S_0_ mode. Nevertheless, our approach illustrates the potential benefits of using MOFBGs as sensors in Lamb wave-based damage detection owing to their ability to discern between the strains recorded in the axial and transverse directions.

**Figure 7:**
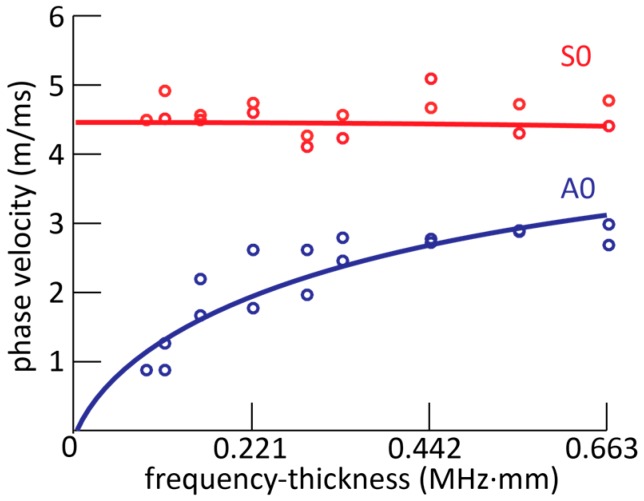
The data points represent the measurement dispersion curve, determined using the MOFBG a Lamb wave sensor in the small, slender CFRP panels. The solid lines indicate the calculated dispersion curves.

Second, the damage can be off the direct path between the actuator and sensors. In such a situation, Lamb waves altered from the damage will yield wave packets arriving at the sensor at different times. In this case, the ability to determine the time-of-arrival of wave packets is crucial [23,24]. Thus, to illustrate the potential of the MOFBGs in time-of-arrival damage detection, we experimentally determined the dispersion curve of the Lamb waves generated in the CFRP panel which also relies on accurate measurements of the time-of-arrival of the wave packets. Recall that the distance between the actuator and sensor is only 10 cm, which is typically insufficient for the A_0_ and S_0_ waves to separate. The result is shown in Figure 7, in which the dots represent the phase velocity determined from the observed time-of-arrival for a certain excitation frequency. We determined the time-of-arrival of both the first wave packet as well as the first reflected wave packet. The solid lines in Figure 7 show the dispersion curves calculated using the material parameters and the panel geometry (detailed in Section 2). We conclude that there is good agreement between the calculated and observed values, as we observed no discrepancies exceeding 10% except the second lowest frequency-thickness tested (at 0.1105 MHz·mm) for the A_0_ mode, which differed by 14%. Using the propagation velocities, we can calculate the distance required for the five-cycle wave packets of A_0_ and S_0_ to separate, which is about 18 cm for the frequency-thickness of 0.221 MHz·mm. 

## 4. Conclusions

In conclusion, we demonstrated, for the first time to our knowledge, the use of MOFBG-based sensors embedded within a composite panel to measure Lamb waves. Until now, mostly surface-mounted FBGs have been used for this purpose. The MOFBGs used in this paper exhibit selective sensitivity to axial and transverse strains, which allows distinguishing between the two fundamental Lamb wave modes. The ability of our sensing scheme to successfully discern between the two fundamental Lamb wave modes offers perspectives for detecting higher order modes as well. In addition, the orientation of the MOFBG (by adjusting the roll angle and aligning the fibre axis ‘S’) allows for the selection of preferential directions of strain. In this manner, directions corresponding to critical locations in a structure can be promoted while undesired reflections impinging from other directions can be impeded. Furthermore, we illustrated in two scenarios the ability to discern the axial and transverse directions with the intention to improve and simplify Lamb wave-based damage detection with the damage located either on or off the direct path between the actuator and sensor. We showed that the ability to make the distinction between the axial and transverse strain directions is clearly beneficial in the former case, as the distinction between the S_0_ and A_0_ waves allowed for the individually evaluation of additional losses and for the observation of partial mode conversion. The latter situation was exemplified by determining the time-of-arrival and associated dispersion of the two fundamental Lamb wave modes for a range of frequencies. Despite the small dimensions of the tested panel, we were able to determine the dispersion curve using an embedded MOFBG with an accuracy essentially exceeding 90%. The results summarised above evidence the added value of using MOFBG sensors in Lamb wave detection-based applications.
